# Sex-Related Differences in Gene Expression Following *Coxiella burnetii* Infection in Mice: Potential Role of Circadian Rhythm

**DOI:** 10.1371/journal.pone.0012190

**Published:** 2010-08-13

**Authors:** Julien Textoris, Leang Heng Ban, Christian Capo, Didier Raoult, Marc Leone, Jean-Louis Mege

**Affiliations:** 1 Unité de Recherche sur les Maladies Infectieuses Tropicales et Emergentes, Centre National de la Recherche Scientifique Unité Mixte de Recherche 6236, Faculté de Médecine, Marseille, France; 2 Service d'anesthésie et de réanimation, Hôpital Nord, Assistance Publique – Hôpitaux de Marseille, Université de la Méditerranée, Marseille, France; National Institute of Allergy and Infectious Diseases, National Institutes of Health, United States of America

## Abstract

**Background:**

Q fever, a zoonosis due to *Coxiella burnetii* infection, exhibits sexual dimorphism; men are affected more frequently and severely than women for a given exposure. Here we explore whether the severity of *C. burnetii* infection in mice is related to differences in male and female gene expression profiles.

**Methodology/Principal Findings:**

Mice were infected with *C. burnetii* for 24 hours, and gene expression was measured in liver cells using microarrays. Multiclass analysis identified 2,777 probes for which expression was specifically modulated by *C. burnetti* infection. Only 14% of the modulated genes were sex-independent, and the remaining 86% were differentially expressed in males and females. Castration of males and females showed that sex hormones were responsible for more than 60% of the observed gene modulation, and this reduction was most pronounced in males. Using functional annotation of modulated genes, we identified four clusters enriched in males that were related to cell-cell adhesion, signal transduction, defensins and cytokine/Jak-Stat pathways. Up-regulation of the IL-10 and Stat-3 genes may account for the high susceptibility of men with Q fever to *C. burnetii* infection and autoantibody production. Two clusters were identified in females, including the circadian rhythm pathway, which consists of positive (Clock, Arntl) and negative (Per) limbs of a feedback loop. We found that Clock and Arntl were down-modulated whereas Per was up-regulated; these changes may be associated with efficient bacterial elimination in females but not in males, in which an exacerbated host response would be prominent.

**Conclusion:**

This large-scale study revealed for the first time that circadian rhythm plays a major role in the anti-infectious response of mice, and it provides a new basis for elucidating the role of sexual dimorphism in human infections.

## Introduction

Social factors such as gender inequity can explain differences in the distribution of infectious diseases between men and women. As shown elsewhere, poor women may be at a disadvantage in their ability to access quality health care [1]. However, biological differences are also responsible for part of the epidemiological variation observed between males and females in infectious diseases due to intra- and extracellular pathogens [2]. Gender-based biological differences also affect host immune responses to pathogens. Women elicit more vigorous humoral and cell-mediated immune responses than men in response to antigenic challenges, while men have frequently been observed to exhibit more aggressive and harmful inflammatory responses to pathogens [3]. Tuberculosis [4], [5] and Legionnaire's disease [6] are reported to be more prevalent and severe in men than in women. Although biological differences have been largely attributed to sex hormones [7], the precise nature of the cross-talk between sex and infection remains largely unknown.

Q fever is a zoonosis due to *Coxiella burnetii*, an intracellular bacterium [8], [9]. Following primary infection, half of infected patients experience acute Q fever. The disease is characterized by clinical polymorphism and includes fever, granulomatous hepatitis, and pneumonia. For a similar exposure risk, men are 2.5 times more likely to become symptomatic than women [10]. Hyperinflammatory Q fever with granulomatous hepatitis and auto-antibodies has been reported in male patients who respond poorly to antimicrobial agents [11]. In addition, Q fever complications are higher in males than in females. As a result, males represent 75% of patients diagnosed with *C. burnetii* endocarditis [12]. In mice, the severity of *C. burnetii* infection is also sex-dependent, with females exhibiting lower bacterial load than males. Ovariectomy increases the bacterial load in the liver and spleen, and this is corrected by 17β-estradiol treatment [13], demonstrating that estrogens limit tissue infection. Nevertheless, the underlying mechanisms that govern the differential responsiveness of males and females to bacterial infection are poorly understood. To investigate these differences, we adopted a large-scale approach consisting of a microarray covering the whole genome. Unexpectedly, 86% (2,379/2,777) of the probes that were modulated by *C. burnetii* infection were dependent on sex. We identified a specific pathway related to the circadian cycle in females that may control the severity of *C. burnetii* infections.

## Results and Discussion

**Figure 1:**
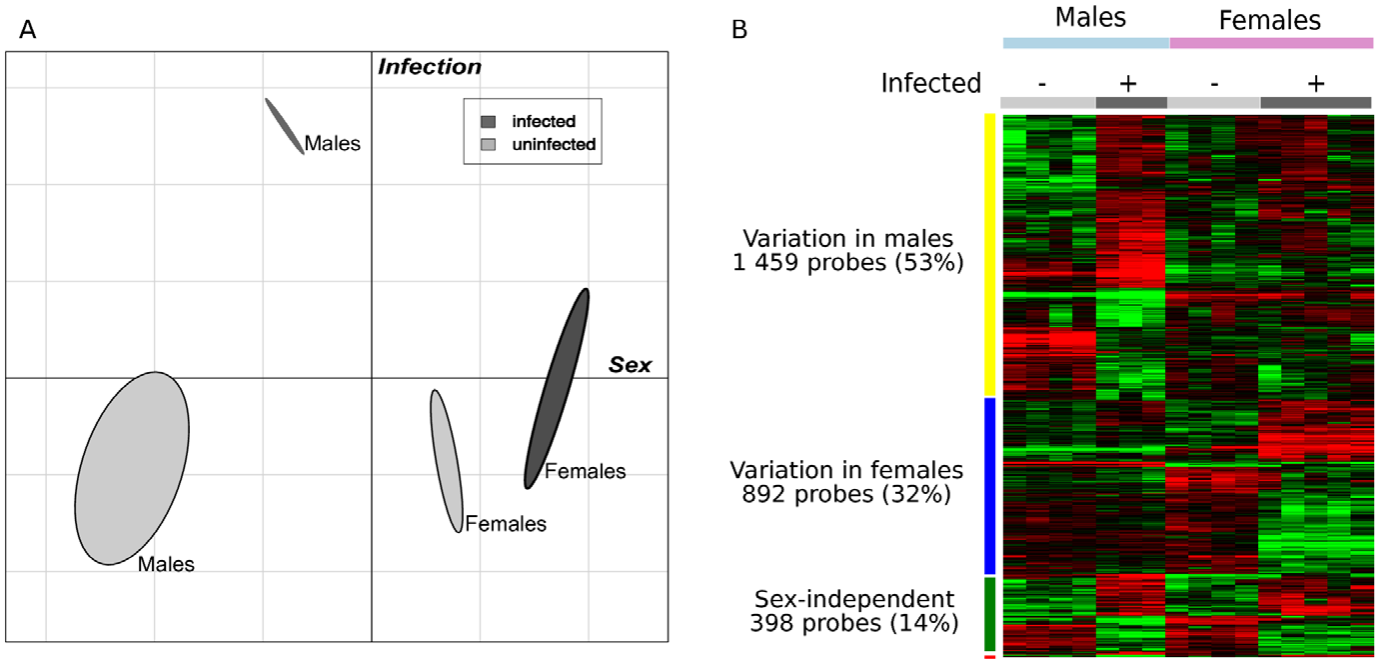
Impact of sex on *C. burnetii* infection. Male and female mice were infected with *C. burnetii* for 24 hours. Transcriptional responses were assessed by microarray. A, The impacts of sex and infection on gene expression were analyzed by principal component analysis using R. Each axis distance represents the amount of variance in gene expression explained by the corresponding factor (sex or infection). B, Hierarchical clustering analysis was used to classify selected up-regulated and down-regulated genes in four clusters: sex-independent genes, male-dependent genes, female-dependent genes and genes inversely modulated in males and females (red vertical bar).

**Figure 2:**
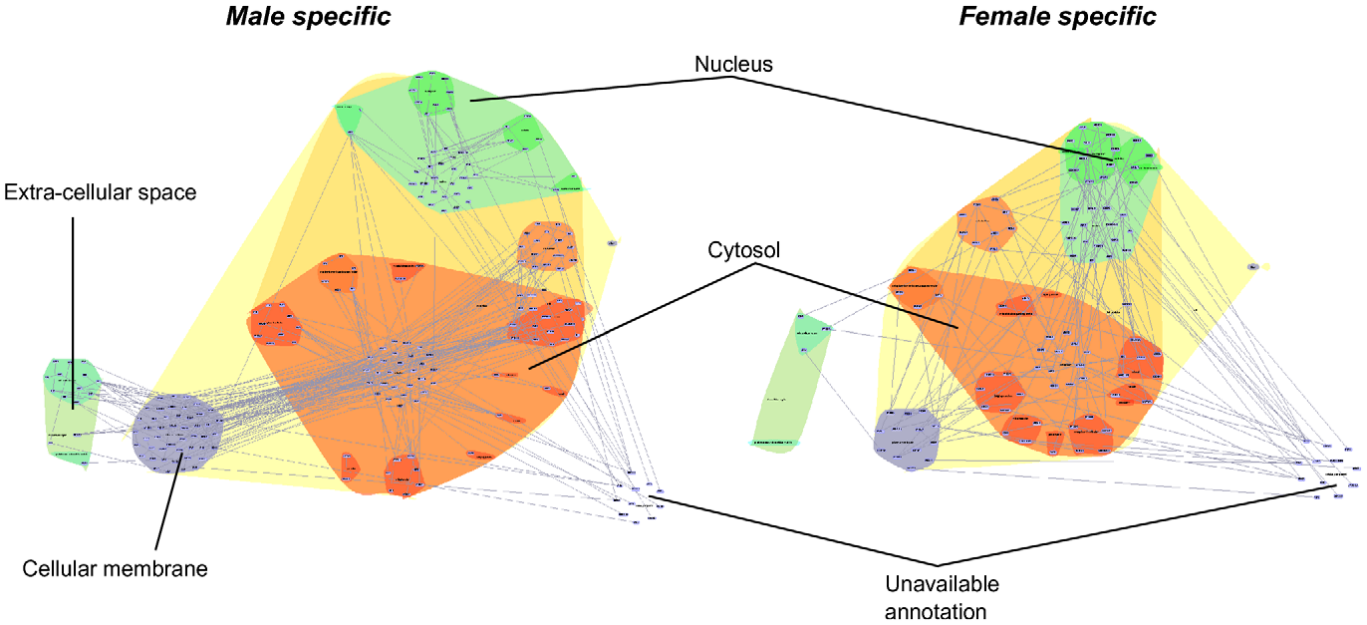
Subcellular distribution of proteins encoded by modulated genes. The InteractomeBrowser plugin of TranscriptomeBrowser was used to generate a graph of protein interactions in males and female mice according to their cellular distribution [65].

Mice were infected with *C. burnetii* for 24 hours, and changes in gene expression were investigated in liver cells. *C. burnetii* infection generated more transcriptional changes in males than in females. When samples were plotted according to the weight of variances due to sex and infection, principal component analysis (PCA) clearly discriminated males and females and revealed that the distance between uninfected and infected males was higher than that found between uninfected and infected females (Fig. 1A). A multiclass analysis identified 2,777 probes that were modulated in response to *C. burnetii*. These probes were classified into four distinct clusters (Fig. 1B). The first cluster is sex-independent and includes 398 probes (14% of the total) that were similarly regulated in both males and females. The other three clusters, representing the majority of probes modulated by *C. burnetii* infection (86% of the total), are sex-dependent: 1,459 probes (53%) were specifically modulated in males whereas 892 (32%) were specifically modulated in females. The fourth cluster of 28 probes is divergent between males and females, with 14 probes up-regulated in males and 14 up-regulated in females (for the list, see Table S1). Using *in silico* analysis, we related the modulated genes to the subcellular distribution of their encoded proteins (Fig. 2). In infected males, the modulated genes encode proteins that have a high degree of interconnection. These interactions spread across the membrane, underlying that a profound reorganization occurred at the membrane after infection. In infected females, there were less interactions between proteins encoded by modulated genes at the membrane level than in males, but the interconnection between the cytosol and the nucleus was apparently more developed. These results highlight important changes in transcription in male and female mice infected with *C. burnetii*.

It is known that nonsexual tissues and cells manifest sex-related differences in most animals including humans. Many of these differences are dependent on sex hormones but it is seems difficult to attribute other differences to hormones [14]. A microarray approach performed on long-lived mice has showed that 381 genes (on over 14,000 genes) are altered in these mice compared to wild type mice in a sex-independent manner while 110 genes are affected in a sex-dependent manner [15]. Our analysis take into account genes modulated in response to *C. burnetii*: these genes are included in two categories, namely sex-independent and sex-dependent genes. Unexpectedly, sex-dependent genes represent 86% of the total number of genes modulated in response to *C. burnetii*. Among them, the majority (80%) were not modulated upon sex in uninfected animals. It is well known that, in humans, the clinical expression of different infectious diseases including tuberculosis [5], Legionnaire's disease [6] and Q fever [10] is associated to sex. Sex has been often described as involved in the incidence of common infectious diseases such as pneumonia [16]. Similar results have been obtained in mice infected with *Listeria monocytogenes* [17] or *Plasmodium berghei* [18]. Bacterial sepsis, a major cause of morbidity and mortality in humans, is due to an exaggerated response of hosts [19]. The frequency and the severity of bacterial sepsis are also associated with sexual dimorphism [2], [20]. Interestingly, such differences are also found in isolated human circulating leukocytes [21] and animal models of endotoxemia [22]. Finally, only few data show that sex does not influence human infections: this has been reported for cerebrospinal [23] and soft tissue [24] infections.

The molecular mechanisms that underlie the differences between males and females in bacterial infections are only in part characterized. As sexual hormones may play a major role in the transcriptional responses to *C. burnetii* infection in male and female mice, we investigated the effect of castration on these responses. Castration shortened the distance between uninfected and infected males, reflecting less pronounced changes in gene expression (Fig. S1A). Castration did not substantially modify the impact of infection on females (Fig. S1B). After *C. burnetii* infection, the number of modulated probes designated as sex-independent remained remarkably constant in castrated mice (396 vs. 398 in intact mice). In contrast, among the sex-dependent probes, only 902 probes were modulated in castrated mice compared with 2,379 probes in intact mice, suggesting that sex hormones were responsible for more than 60% of the gene modulation (Fig. S2). This reduction was most significant in males: 378 probes were specifically modulated in castrated males (vs. 1,459 in intact males) compared to 516 in castrated females (vs. 892 in intact females), suggesting that testosterone was responsible for the major changes observed after *C. burnetii* infection. This finding is in line with previous studies suggesting the predominant effect of testosterone on sexual dimorphism [25], [26]. It is important to note that about 40% of sex-dependent genes that were modulated in response to *C. burnetii* were not linked to hormonal status. This is reminiscent of previous data that showed that sex differences are not completely abrogated or accentuated by hormone ablation or supplementation, respectively [27].

We next used functional annotation of the modulated genes to identify major pathways that were possibly involved in the response to *C. burnetii* infection. In the sex-independent cluster, four groups of enriched keywords were identified (Table 1). Group 1, with an enrichment score of 4.3, is related to early induction of the acute phase liver response. Acute phase response is a major function of the liver that contributes to bacterial clearance in acute Q fever [10]. The gene encoding serum amyloid A1–3 was expressed with a fold change (FC) of over 25. The genes encoding orosomucoid 1–3, serine peptidase inhibitor and complement components (C8a, C9) were also up-regulated. Group 2 (enrichment score of 3.9) is related to lipid metabolism and group 3 (enrichment score of 3.3) is related to steroid metabolism. Most of the genes annotated with keywords of groups 2 and 3 were down-regulated by a mean FC of 2. This finding is apparently related to disturbed lipid metabolism during *C. burnetii* infection since pronounced lipid infiltration and increased cholesterol have been reported in the livers of *C. burnetii*-infected guinea pigs [28]. In addition, *C. burnetii* replication requires free access to host cholesterol stores [29]. Group 4 is related to genes encoding proteins with a thrombospondin domain. Thrombospondin is a family of multifunctional proteins involved in coagulation, angiogenesis, apoptosis, and immune regulation [30]. One of these genes (Spon2) is a pathogen-associated molecular pattern recognition molecule known to recognize *Escherichia coli* lipopolysaccharide and numerous gram negative bacteria [31]. Although some partners of thrombospondin such as αvβ3 integrin have been described as *C. burnetii* receptors [32], there is no evidence that Spon2 recognizes *C. burnetii*.

**Table 1:** Functional annotation of the sex-independent cluster.

Ontology	Keywords	*P*-value
**Acute phase response (ES : 4.3–25 genes)**SAA1, SAA2, SAA3, SAA4, ORM3, SERPINA3N, C8a, C9		
*SP_PIR_KEYWORDS*	Acute phase	2.9×10^−6^
*GO_BP*	Acute-phase response	6.4×10^−6^
*GO_BP*	Acute inflammatory response	1.8×10^−5^
*INTERPRO*	Serum amyloid A protein	8.6×10^−5^
*SMART*	SAA	6.1×10^−6^
**Lipid metabolism (ES : 3.9–38 genes)**CRAT, ACOX1, ACOT2, ACOT5, EHHADH, ACAA1A, CPT1B, PLTP		
*GO_BP*	Fatty acid metabolic process	1.3×10^−7^
*GO_BP*	Monocarboxylic acid metabolic process	9.1×10^−9^
*SP_PIR_KEYWORDS*	Fatty acid metabolism	8.4×10^−6^
*BO_BP*	Lipid metabolic process	1.6×10^−9^
*GO_CC*	Peroxysome	3.3×10^−5^
*GO_CC*	Microbody	3.3×10^−5^
**Steroid metabolic process (ES : 3.3–14 genes)**STAR, IDI1, MOGAT1, LEPR, FABP5, PCTP, AKR1C18, FBP2, HMGCS1		
*GO_BP*	Steroid metabolic process	1.7×10^−4^
*GO_BP*	Alcohol metabolic process	5.5×10^−4^
*GO_BP*	Cholesterol metabolic process	6.8×10^−4^
*GO_BP*	Sterol metabolic process	1.0×10^−3^
**Thrombospondin (ES : 1.8–5 genes)**SPON2, C9, SEMA5B, ADAMTS20, C8A		
*SMART*	TSP1	1.7×10^−3^
*INTERPRO*	Thrombospondin, type I	1.9×10^−3^

The enrichment score (ES), the number of genes and example genes in each group of genes were indicated.

**Figure 3:**
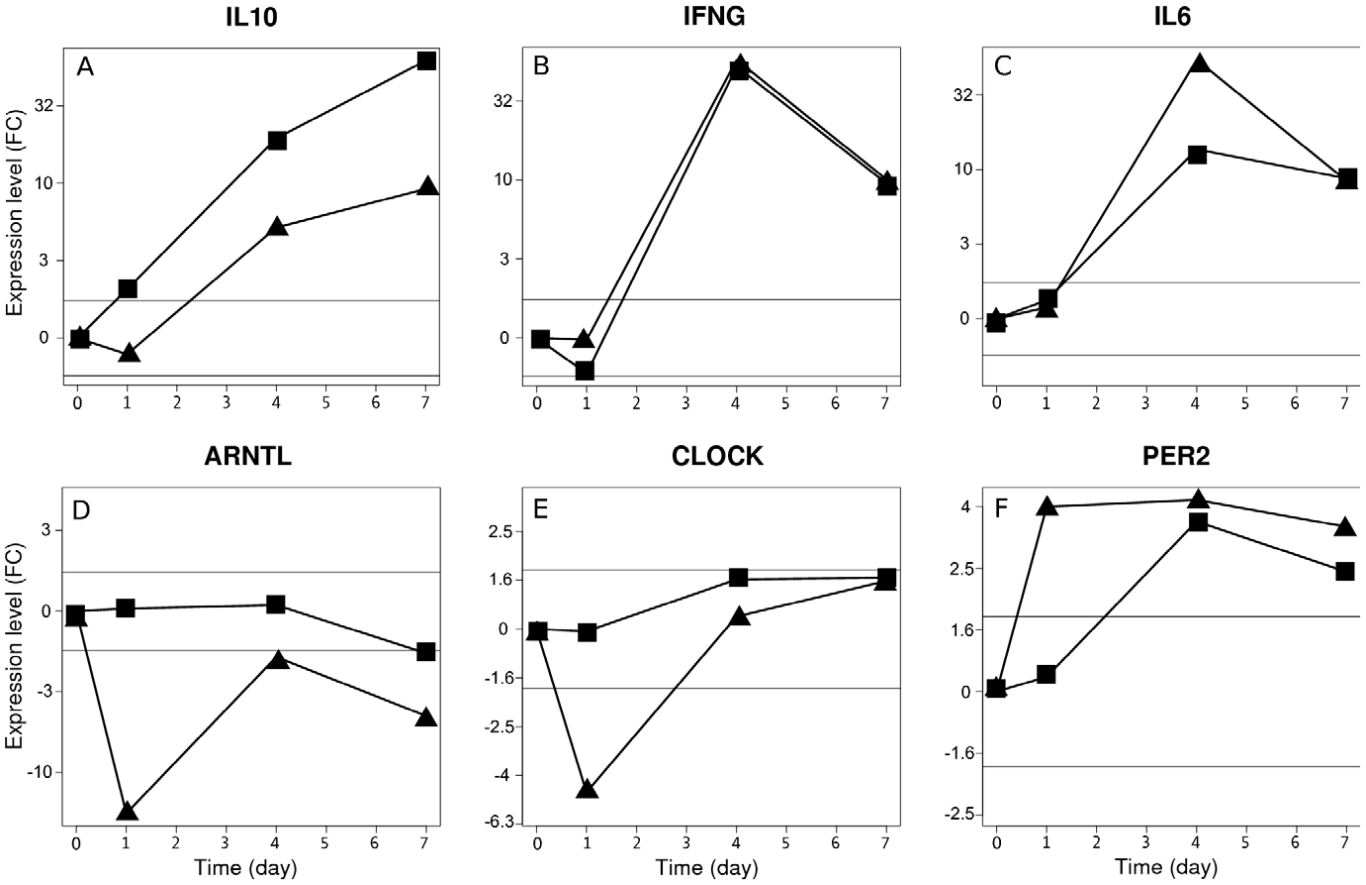
Time course of gene modulation. Male (squares) and female (triangles) mice were infected with *C. burnetii* for one, four and seven days, and gene expression was determined by qRT-PCR and normalized with the GAPDH gene. A–C, genes encoding cytokines. D–F, genes involved in circadian rhythm. Horizontal lines indicate the selected fold change cut-off of ±1.75.

Functional annotation of genes that were modulated only in infected males identified four groups of keywords (Table 2). Groups 1 and 3 are related to signal transduction and cell-cell adhesion, respectively. Keyword group 2 is related to genes encoding defensins. Although defensins are peptides with antimicrobial properties [33], [34], their role against *C. burnetii* infection has never been reported. Defensins such as Defcr3, Hamp1 and Hamp2 were down-regulated in infected males, suggesting that the reduced expression of defensins favors early infection in males. Hepcidins (Hamp1 and Hamp2) reduce iron availability for invading microorganisms by reducing extracellular iron concentrations [35]. As has been recently suggested, greater iron availability in males may favor *C. burnetii* replication [36]. It has also been shown that hepcidins are differentially expressed in male and female mice [37], [38]. Keyword group 4 is related to cytokines and the Jak/Stat pathway. The gene encoding interleukin (IL)-10 was up-regulated in males. These results were confirmed by RT-PCR (Table S2), and a time course study showed that males overproduced interleukin (IL)-10 mRNA compared with females independently of the duration of infection (Fig. 3A). Note that the expression of the gene encoding Stat 3, a transcription factor known to be targeted by IL-10, was higher in males than in females. We previously showed that IL-10 enables monocytes to support *C. burnetii* replication [39], [40] and that chronic evolution of Q fever is associated with increased production of IL-10 [41]. However, to our knowledge, a link between IL-10 and sexual dimorphism in *C. burnetii* infection has never been reported. Interferon (IFN)-γ, which is critical for protection against intracellular bacteria and *C. burnetii* infection [42], was weakly modulated in male and female mice one day after infection as determined by microarray and RT-PCR (Table S2). A time course analysis revealed that the gene encoding IFN-γ was dramatically up-regulated at days 4 and 7 post-infection (Fig. 3B), suggesting that IFN-γ induces deleterious effects in parallel with the resolution of *C. burnetii* infection. The expression of the IL-6 gene was slightly up-regulated in females (Table S2), and a time course study showed that the IL-6 gene was dramatically over-expressed at days 4 and 7 post-infection, especially in females (Fig. 3C). After trauma-hemorrhage, IL-6 release by macrophages is suppressed in male mice but not in female mice [43], suggesting that sex and IL-6 production are related. Taken together, these results suggest that males develop an early anti-inflammatory response that may favor increased bacterial burden at the onset of infection and may promote antibody production in an IL-10-dependent manner. This may account for the presence of auto-antibodies in men with Q fever.

**Table 2:** Functional annotation of male-dependent cluster.

Ontology	Keywords	*P*-value
**Signal transduction (ES : 9.9–418 genes)**EXTL1, CDH11, RHO, ADRA2B, IL12RB2, CD36 GLP2R, OLFR981		
*SP_PIR_KEYWORDS*	Transmembrane	1.2×10^−9^
*GO_BP*	Signal transduction	3.9×10^−7^
*GO_MF*	Transmembrane receptor activity	1.7×10^−9^
*GO_BP*	G-protein coupled receptor signal transduction	4.1×10^−7^
*INTERPRO*	Olfactory receptor	8.8×10^−8^
*GO_GC*	Transmembrane region	2.0×10^−6^
**Defensin (ES : 8.2–45 genes)**DEFB3, DEFA1, DEFCR7, DEFCR17, DEFCR9, DEFCR25, HAMP2		
*SMART*	DEFSN	5.7×10^−11^
*INTERPRO*	Mammalian defensin	1.7×10^−10^
*GO_BP*	Defense response to bacterium	2.2×10^−8^
*SP_PIR_KEYWORDS*	Antimicrobial	9.4×10^−7^
*GO_BP*	Defense response	1.4×10^−3^
*SP_PIR_KEYWORDS*	Defensin	1.2×10^−6^
**Cell-cell adhesion (ES : 3.7–52 genes)**CDH1, CDH4, FAIM2, MMD2, PCDHA5, PCDHB19, CDH11		
*INTERPRO*	Cadherin	2.9×10^−7^
*SP_PIR_KEYWORDS*	Cell adhesion	2.8×10^−4^
*GO_BP*	Cell adhesion	2.5×10^−3^
*SP_PIR_KEYWORDS*	Calcium	3.0×10^−3^
**Cytokines/Jak-STAT pathway (ES : 2.3–28 genes)**IFNA1, IL5, PDGFA, IL1RN, STAT3, EGF,CXCL12, IL10, IL11, IFNG, IL13		
*BIOCARTA*	Cytokines and Inflammatory response	2.6×10^−4^
*KEGG_PATHWAY*	Jak-STAT signalling pathway	1.7×10^−3^
*KEGG_PATHWAY*	Cytokin-Cytokin receptor interaction	4.2×10^−3^
*INTERPRO*	Four-helical cytokine, core	7.1×10^−3^
*SP_PIR_KEYWORDS*	Cytokine	2.1×10^−2^
*GO_MF*	Cytokine activity	2.0×10^−2^

The enrichment score (ES), the number of genes and example genes in each group of genes were indicated.

The specific signature of infected females, which includes 892 genes (Fig. 1B), was characterized by two major groups of keywords (Table 3): intracellular location and transcriptional activity, and unexpectedly, circadian rhythm. The latter pathway was further investigated with RT-PCR and network analysis. Circadian rhythm is controlled by an auto-regulated transcription-translation feedback loop that regulates the expression of rhythmic genes in a tissue-specific manner [44]. In liver cells, a significant number of clock-regulated genes are associated with the cell cycle and proliferation. Clock and Arntl form the positive limb of the transcriptional loop, while the Per and Cry protein families form the negative limb of the feedback loop by inhibiting their own Clock/Arntl-induced transcription [44]. The turnover of Per and Cry allows the cycle to begin again (Fig. S3). Microarray and RT-PCR data showed that Clock and Arntl expression were down-regulated in females infected with *C. burnetii* for one day while Per expression was up-regulated (Table S2). We questioned whether these variations were persistent because we had previously found that *C. burnetii* infection of liver cells peaks after four days and decreases thereafter [13]. The levels of Arntl and Clock transcripts remained constant in males over seven days, but in females, these transcripts declined at day one and increased thereafter to approach the number observed in males (Fig. 3D and 3E, respectively). The expression of Per2, which increased in females at day one, remained high during the seven days of the infection. In males, the expression of the Per2 gene increased at day four (Fig. 3F). The differences between males and females regarding the expression of Arntl, Clock and Per2 were lost after castration of the mice (Fig. S4). Taken together, these findings show that *C. burnetii* infection affected the circadian rhythm of female mice. These results are distinct from those shown in a recent report in which the expression of the Clock, Arntl and Per genes was down-modulated in blood leukocytes after the administration of endotoxin to human volunteers. However, the relationship between these changes and sexual dimorphism was not investigated [45].

**Table 3:** Functional annotation of female-dependent cluster.

Ontology	Keywords	*P*-value
**Circadian rhythm (ES : 2.8–14 genes)**CLOCK, PER1, PER2, PER3, PTGDS, OPN3, MTNR1A, ARNTL, AFP		
*GO_BP*	Circadian rhythm	2.9×10^−8^
*GO_BP*	Rhythmic process	1.0×10^−6^
*SP_PIR_KEYWORDS*	Biological rhythms	2.0×10^−6^
*KEGG_PATHWAY*	Circadian rhythm	3.6×10^−5^
*INTERPRO*	PAS domain	8.5×10^−4^
**Intracellular/Transcription (ES : 2.2–328 genes)**SOX9, PBEF1, STX1B1, RNF20, UGT1A2		
*GO_CC*	Intracellular	3.5×10^−5^
*GO_CC*	Membrane-bound organelle	2.5×10^−3^
*GO_CC*	Nucleus	7.3×10^−3^
*INTERPRO*	Basic helix-loop-helix dimerization region	2.0×10^−3^
*GO_BP*	Metabolic process	2.8×10^−4^

The enrichment score (ES), the number of genes and example genes in each group of genes were indicated.

The circadian rhythm modification associated with *C. burnetti* infection may affect numerous biological functions. The biological clock controls the secretion of estrogen hormones [46] by regulating the intracellular levels of estrogen receptors α and β [47], [48]. Lower Per2 levels lead to the stabilization of estrogen receptor β. As Per2 is estrogen-inducible, a feedback mechanism may attenuate stimulation by estrogens [48]. Male steroid hormones retroactively play a role in the circadian rhythm at the brain level [49]. Orchidectomy leads to a loss of androgen receptor expression and circadian-specific locomotor activity, both of which are restored by testosterone replacement [49], [50]. Interestingly, circadian rhythm and immune responses are interconnected [51]. For instance, the susceptibility or resistance of flies to infection varies according to the time when they are infected [52]. The circadian oscillation of clock genes affects IFN-γ production through the regulation of Per2 [53] or the activity of natural killer cells [54]. Circadian rhythm may also control the steroid pathway since it post-translationally inhibits glucocorticoid receptors [55]. Cross-talk between these pathways may reinforce the effect of sex on *C. burnetii* infection. Finally, we hypothesize that an early response involving circadian rhythm likely promotes efficient bacterial elimination in females, while in males, the early phase of the response to *C. burnetii* infection is inefficient, due at least in part to an anti-inflammatory response that favors bacterial growth [13].

In conclusion, different transcriptional responses to *C. burnetii* were associated with the severity of the infection in male and female mice. Functional annotation showed that the modulated genes were organized in different networks in males and females and we hypothesize that circadian rhythm may be involved in *C. burnetii* infection. As the percentage of sex-dependent genes that were modulated by *C. burnetii* infection was dramatically high, one can expect that numerous genes would be modulated in other infectious diseases described as epidemiologically associated with sexual dimorphism. Our data provide a new basis for elucidating the role of sexual dimorphism in human infections. It would be also interesting to determine if differences in gene expression occur in infectious diseases not described as epidemiologically sex-dependent.

## Materials and Methods

### Infection of Mice

The following experimental protocol was approved by the Institutional Animal Care and Use Committee of the Université de la Méditerranée. Forty C57BL/6 mice (20 males and 20 females) were obtained from Charles River Laboratories. Ten females and ten males were sterilized at seven weeks of age. Ovaries were removed through bilateral incisions of the lumbar region and testes were removed through bilateral scrotum incision. After three weeks, ten males and ten females, including five non-castrated and five castrated animals of each gender, were killed. The other series of mice were injected intraperitoneally with 10^5^ *C. burnetii* organisms at the same time and sacrificed at day one after infection [56]. In another set of experiments, 16 mice (4 intact males, 4 castrated males, 4 intact females and 4 castrated females) were killed at zero, one, four and seven days after *C. burnetii* infection. Organs were aseptically excised. Samples for RNA extraction were stabilized in RNA*later* (Qiagen, France). All specimens were stored at −80°C until use.

### RNA isolation and real-time PCR

Total RNA extraction and reverse transcription were performed as previously described [57]. The primers were designed by Primer3 (v. 0.4.0) available at http://frodo.wi.mit.edu/. Reverse trancriptase was omitted in negative controls. The FC in target gene cDNA relative to endogenous control (glyceraldehyde 3-phosphate dehydrogenase, GAPDH) was determined with the formula: fold change = 2^−ΔΔCt^, where ΔΔCt = (Ct_Target_−Ct_GAPDH_)_infected mice_−(Ct_Target_−Ct_GAPDH_)_uninfected mice_. Ct values were defined as the cycle number at which fluorescence signals were detected [58].

### Microarray procedures and data analysis

Whole Mouse Genome Oligo Microarray 4×44K Kit (44,000 60-mer oligonucleotides) and One-Color Microarray-Based Gene Expression Analysis (Agilent Technologies) were used as recently described [57]. Slides were washed, dried, and scanned at 5-µm resolution with a G2505B DNA microarray scanner (Agilent Technologies). Intensity signals were generated using the Agilent Feature Extractor Software A.9.1.3 after image quality control was performed manually. Four aberrant samples (two infected males, one uninfected male and one uninfected female) were eliminated before starting the analysis because of technical problems. Raw signal data were normalized with the quantile method [59] and then transformed into binary logarithm. The Significance Analysis of Microarrays test (SAM [60]) was used to study the gene expression in intact animals with a multiclass analysis (sex and infection). False Discovery Rate (FDR) was set to 0.05 to filter modulated genes. Then, selected probes (n = 13,814) were filtered using the FC obtained from dividing the median intensity of infected and non-infected mice. FCs of male and female mice were calculated with a cut-off at 1.75 as selection criterium for differentially expressed genes. Thus, the selected genes which were annotated as “sex-specific” were also “infection-specific”. Supervised analyses were carried out with R (R for statistical computation and graphic version 2.7.2, GPL) with the library BioConductor [61]. Functional annotations and classifications were performed using the DAVID Bioinformatics Resource 2008 [62]. Keywords from the following ontologies were analyzed (GO, SP_PIR_KEYWORDS, UP_SEQ_FEATURE, SMART, INTERPRO, KEGG_PATHWAYS, and BIOCARTA). Gene Ontology (GO) keywords are further classified according to their main category: Biological Process (GO_BP), Cellular Component (GO_CC) or Molecular Function (GO_MF). PCA was used to visually explore global effects for genome wide trends, unexpected effects and outliers in the expression data (library made4 for R [63]). The data discussed in this publication have been deposited in NCBI's Gene Expression Omnibus [64] and are accessible through GEO Series accession number GSE21065 (http://www.ncbi.nlm.nih.gov/geo/query/acc.cgi?acc=GSE21065).

## Supporting Information

Figure S1 Impact of castration on *C. burnetii* infection. Male and female mice were castrated and then infected with *C. burnetii* for 24 hours. Transcriptional responses were assessed by microarray. The impacts of castration and infection on gene expression were assessed by principal component analysis in males (A) and females (B) using R. Each axis distance represents the amount of variance in gene expression explained by the corresponding factor (castration or infection).
(7.19 MB TIF)

Figure S2 Castration reduces the number of genes regulated by infection in males. Genes modulated by *C. burnetii* infection in sterilized or intact male and female mice are represented by a Venn diagram.
(1.46 MB TIF)

Figure S3 Overview of the tissular components of the biological clock. The heterodimer Clock/Arntl positively regulates transcription of the Per and Cry genes, which in turn negatively regulate Clock and Arntl transcription, creating cyclic expression of these proteins.
(1.78 MB TIF)

Figure S4 Time course of circadian gene modulation in castrated animals after infection. Castrated male (squares) and ovariectomized female (triangles) mice were infected with *C. burnetii* for one, four and seven days, and gene expression was determined by qRT-PCR and normalized to the GAPDH gene. A–C, Genes involved in circadian rhythm. Horizontal lines indicate the selected fold change cut-off of ±1.75.
(4.08 MB TIF)

Table S1 Inversed fold changes in males and females.
(0.05 MB PDF)

Table S2 Fold changes assessed by microarray and RT-PCR.
(0.04 MB PDF)
