# Supplementary figures and images for: Sex-Related Differences in Gene Expression Following Coxiella burnetii Infection in Mice: Potential Role of Circadian Rhythm
Source: PLoS One. 2010 Aug 13;5(8):e12190. doi: 10.1371/journal.pone.0012190 (PMC2921390; doi:10.1371/journal.pone.0012190)

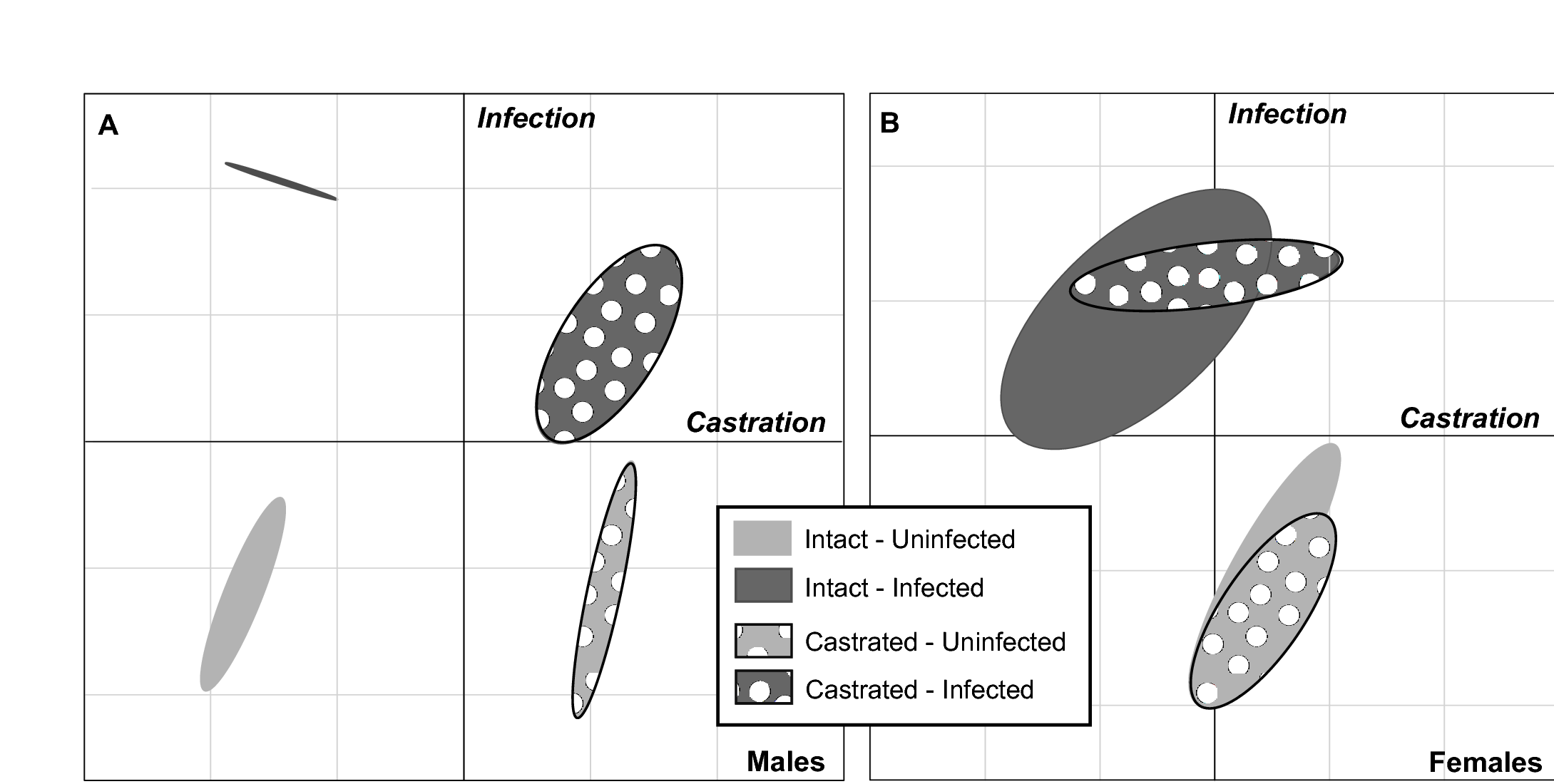

Supplement: Figure S1 — Impact of castration on C. burnetii infection. Male and female mice were castrated and then infected with C. burnetii for 24 hours. Transcriptional responses were assessed by microarray. The impacts of castration and infection on gene expression were assessed by principal component analysis in males (A) and females (B) using R. Each axis distance represents the amount of variance in gene expression explained by the corresponding factor (castration or infection). (7.19 MB TIF) [file pone.0012190.s001.tif]

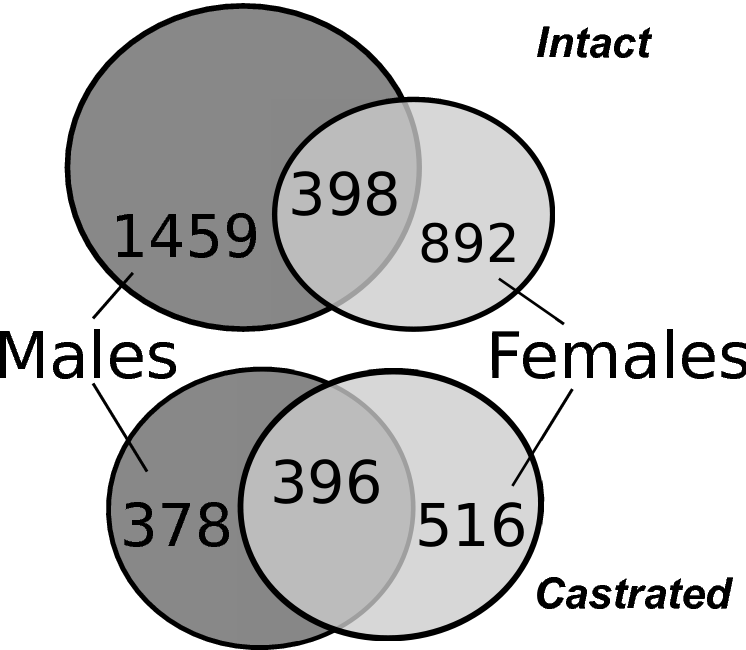

Supplement: Figure S2 — Castration reduces the number of genes regulated by infection in males. Genes modulated by C. burnetii infection in sterilized or intact male and female mice are represented by a Venn diagram. (1.46 MB TIF) [file pone.0012190.s002.tif]

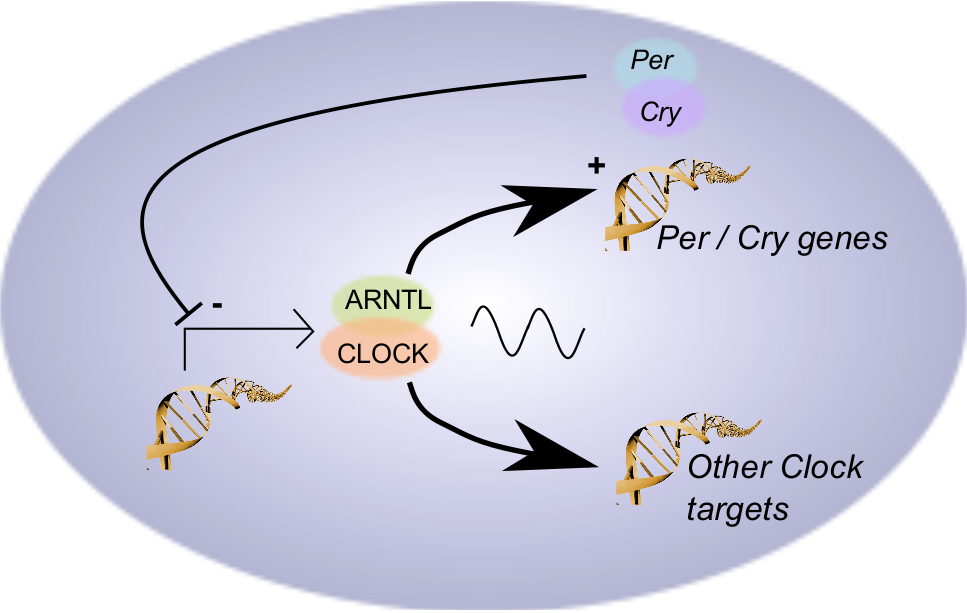

Supplement: Figure S3 — Overview of the tissular components of the biological clock. The heterodimer Clock/Arntl positively regulates transcription of the Per and Cry genes, which in turn negatively regulate Clock and Arntl transcription, creating cyclic expression of these proteins. (1.78 MB TIF) [file pone.0012190.s003.tif]

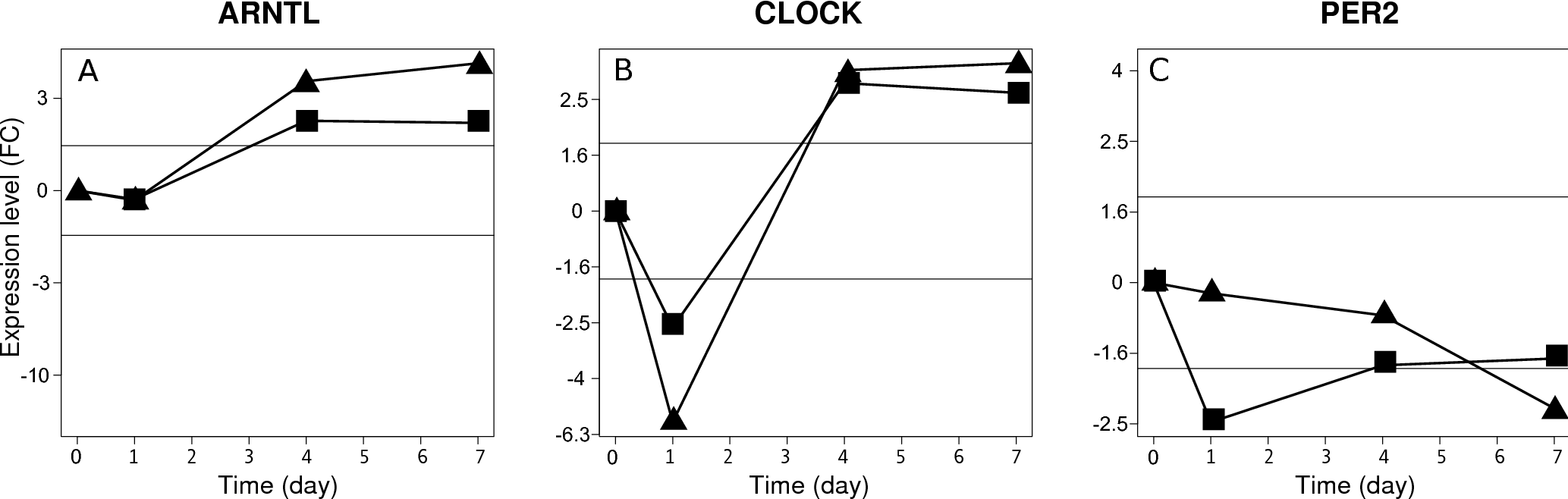

Supplement: Figure S4 — Time course of circadian gene modulation in castrated animals after infection. Castrated male (squares) and ovariectomized female (triangles) mice were infected with C. burnetii for one, four and seven days, and gene expression was determined by qRT-PCR and normalized to the GAPDH gene. A–C, Genes involved in circadian rhythm. Horizontal lines indicate the selected fold change cut-off of ±1.75. (4.08 MB TIF) [file pone.0012190.s004.tif]
