# Supplementary material for: Sex-Related Differences in Gene Expression Following Coxiella burnetii Infection in Mice: Potential Role of Circadian Rhythm
Source: PLoS One. 2010 Aug 13;5(8):e12190. doi: 10.1371/journal.pone.0012190 (PMC2921390; doi:10.1371/journal.pone.0012190)
Supplement: Table S1 — Inversed fold changes in males and females. (0.05 MB PDF) [file pone.0012190.s005.pdf]

Table S1. Inversed fold changes in males and females

down-modulated in males up-regulated in females			up-regulated in males down-modulated in females		
BB505010	- 12.5	2.6	D930050A07Rik	1.8	- 2.0
Gm534	- 6.7	1.9	PPP1R3C	1.8	- 2.2
AK007854	- 5.6	1.9	CHKA	1.9	- 2.6
LHX8	- 3.1	1.9	BAIAP2	1.9	- 2.7
CYP3A44	- 2.4	2.7	MLL5	1.9	- 2.0
AACS	- 2.3	2.8	MYBPC2	1.9	- 2.2
RGS16	- 2.2	11.5	FST	1.9	- 1.8
AK051762	- 2.1	1.8	CSPG5	2.0	- 2.7
OPRM1	- 2.0	1.9	BC057022	2.1	- 1.9
6030468B19Rik	- 2.0	1.9	AK040776	2.3	- 1.9
Gm7231	- 1.9	3.4	LOXL4	2.4	- 4.3
OLFR1321	- 1.9	2.3	EMID2	2.9	- 1.8
BAI3	- 1.8	1.9	COG4	3.2	- 1.8
Gm10001	- 1.8	2.0	OLFR914	3.8	- 3.0
