# Supplementary material for: Sex-Related Differences in Gene Expression Following Coxiella burnetii Infection in Mice: Potential Role of Circadian Rhythm
Source: PLoS One. 2010 Aug 13;5(8):e12190. doi: 10.1371/journal.pone.0012190 (PMC2921390; doi:10.1371/journal.pone.0012190)
Supplement: Table S2 — Fold changes assessed by microarray and RT-PCR. (0.04 MB PDF) [file pone.0012190.s006.pdf]

Table S2. Fold changes assessed by microarray and RT-PCR

	Fold change			
	**microarray**		**RT-PCR**	
	males	females	males	females
IL6	1.3	2.2	1.3	1.2
IFNG	- 1.1	- 1.1	- 1.7	1.0
IL10	1.7	1.2	2.1	1.2
ARNTL	1.4	- 11.1	1.0	-18
CLOCK	1.1	- 2.6	1.0	- 4.7
PER2	1.2	3.91	1.1	4.0
